# Ami - The chemist's amanuensis

**DOI:** 10.1186/1758-2946-3-45

**Published:** 2011-10-14

**Authors:** Brian J Brooks, Adam L Thorn, Matthew Smith, Peter Matthews, Shaoming Chen, Ben O'Steen, Sam E Adams, Joe A Townsend, Peter Murray-Rust

**Affiliations:** 1 Unilever Centre for Molecular Science Informatics, Department of Chemistry, University of Cambridge, Lensfield Road, Cambridge CB2 1EW, UK

## Abstract

The Ami project was a six month Rapid Innovation project sponsored by JISC to explore the Virtual Research Environment space. The project brainstormed with chemists and decided to investigate ways to facilitate monitoring and collection of experimental data.

A frequently encountered use-case was identified of how the chemist reaches the end of an experiment, but finds an unexpected result. The ability to replay events can significantly help make sense of how things progressed. The project therefore concentrated on collecting a variety of dimensions of ancillary data - data that would not normally be collected due to practicality constraints. There were three main areas of investigation: 1) Development of a monitoring tool using infrared and ultrasonic sensors; 2) Time-lapse motion video capture (for example, videoing 5 seconds in every 60); and 3) Activity-driven video monitoring of the fume cupboard environs.

The Ami client application was developed to control these separate logging functions. The application builds up a timeline of the events in the experiment and around the fume cupboard. The videos and data logs can then be reviewed after the experiment in order to help the chemist determine the exact timings and conditions used.

The project experimented with ways in which a Microsoft Kinect could be used in a laboratory setting. Investigations suggest that it would not be an ideal device for controlling a mouse, but it shows promise for usages such as manipulating virtual molecules.

## Background

*Amanuensis: One employed to take dictation, or copy manuscripts; A clerk, secretary or stenographer, or scribe *http://en.wiktionary.org/wiki/amanuensis

**Figure 1:**
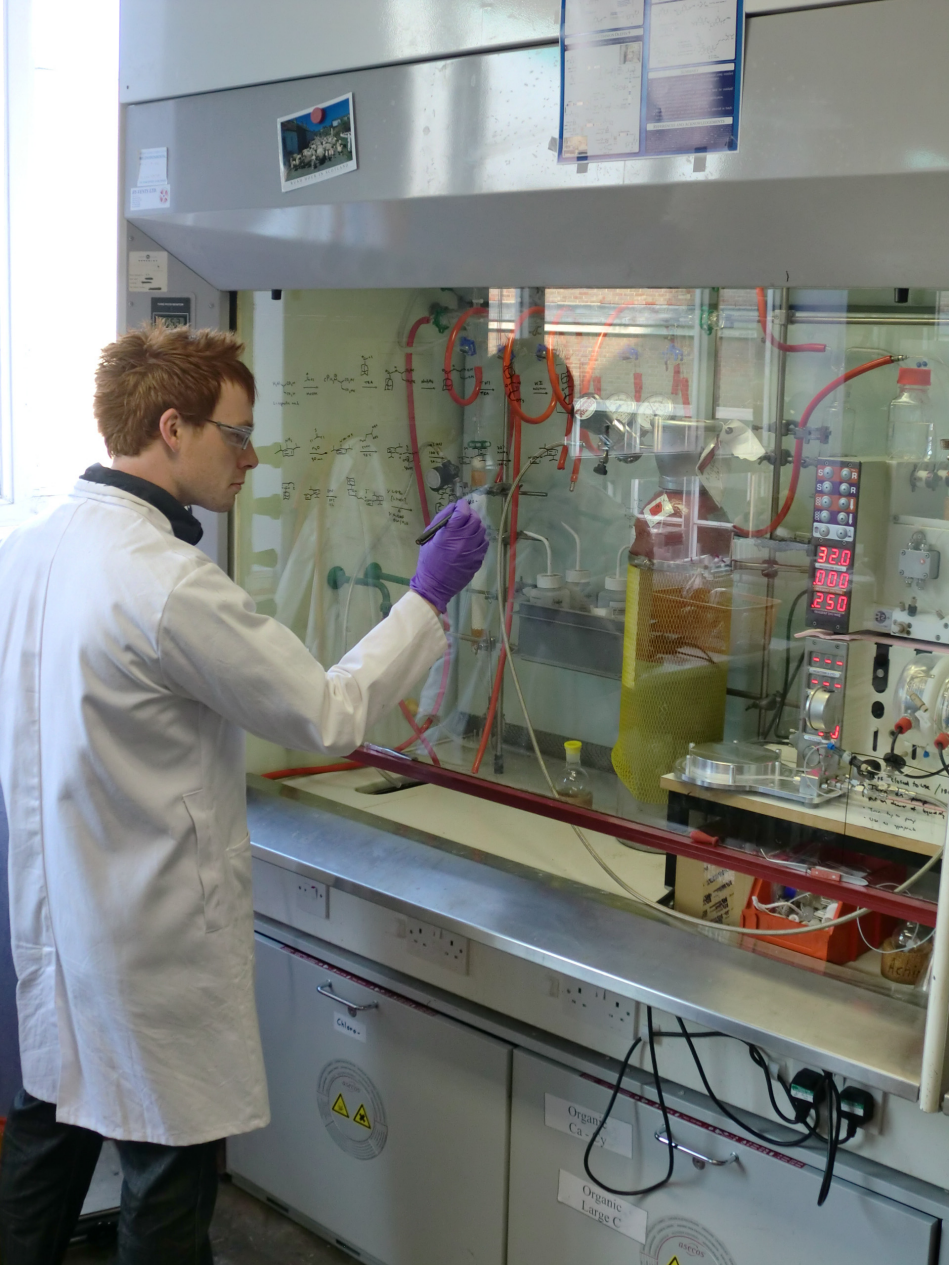
**Chemist working at a typical fume cupboard**. It is common to use the glass front for drawing reactions, jotting notes, etc.

**Figure 2:**
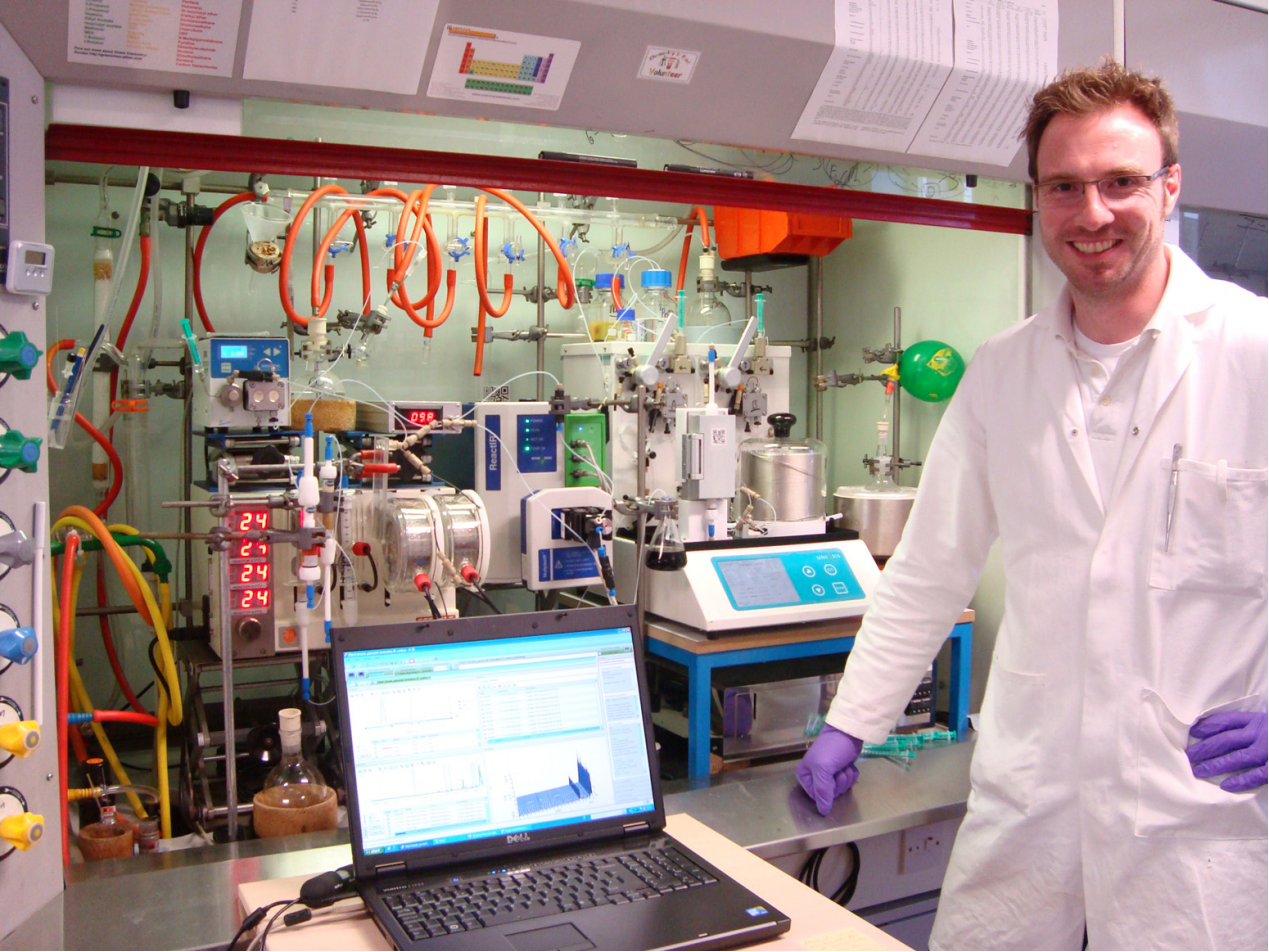
**A chemist happy in his element, and probably with his elements too**. Note the protective clothing. Space in the lab is at a premium; catering for computer is an after-thought.

The chemistry laboratory is a difficult environment for using a computer. Space is at a premium; benches and fume cupboards are covered with apparatus and typically have chemicals that are detrimental to computers (Figure 1). The chemist wears protective clothing, and often has gloves on (Figure 2). Lots of little issues add up to make it a challenge to successfully use computers in the lab.

Yet the collection of data has never been more important. Trends in science are to require data in support of experimental results. It is considered that research paid for by public money should have the proceeds visible to anyone who may wish to use them. Recent examples of challenging the conclusions of the scientific community - such as MMR episode [1] or the Climate-Gate emails [2] - plus various examples of scientific fraud, are all events that could have been ameliorated if their data were open for review.

In recent years, various projects have highlighted how hardware and software can be used for the collection, management and use of laboratory information. At the University of Cambridge Cavendish Laboratory Baumberg *et al*. [3] illustrate how new hardware forms (desktop "Surfaces" and tablets) facilitate the use of visual sketching techniques to enhance the scientific process, in particular within a group. The Frey group at Southampton have shown [4,5] how semantic tools can be used to link complex information from the whole experiment lifecycle. The OPSIN chemical name-to-structure program [6,7] developed by Lowe *et al*. here in the Unilever Centre has been extended to show how complex information can be accessed using smartphones [8].

The Ami project was created to find improved ways for chemists to use computers in the lab. The goal was to build a prototype next-generation information assistant "natural user interface" for scientists working at the lab bench. The limitations of paper lab notebooks are well recorded, and the Chemistry Department at Cambridge has recently deployed a commercial electronic lab notebook (ELN), a project in which one of the Ami team members was a significant participant. The Ami project aimed to combine and develop existing hardware and software technologies in novel ways to provide an information rich environment for the scientist at the bench.

### Methodology

The Ami project used a brainstorming session with chemists from the department plus representatives from the Chemistry Department's computing service to identify the issues that chemists have to deal with and how computers could be used to address them (see Appendix 1 for further details). A common use-case that emerged was the example of how chemists often reach the end of their experiment and find an unexpected result. Often the suspicion is that the unexpected result could be due to mundane reasons; the conditions used for the reaction may have varied unexpectedly, or reaction components were not added, or timing was critical, or the wrong chemicals were used, etc. What was required was some way of going back over events to see what actually did happen. What was needed was some way of collecting ancillary data - data that is not the primary data that is scientifically obvious to collect - that could be consulted after the experiment is finished if circumstances needed to be investigated further.

The desire to log ancillary data identified three areas to work on. The first was to build some hardware device that could monitor parameters such as the temperature of the reaction vessel, keeping a log over the whole duration of the experiment. The second was a video monitor to provide a close-up visual record of the reaction. The third area was a wide-angle video monitor of the whole fumehood which would log all activity in the vicinity of the reaction.

Windows 7 was chosen as the development platform for Ami. This was because of the wide availability of software tools and utilities available for Windows, and also because of the experience within the group. Where possible code was developed in Java, using the IntelliJ IDEA development tool.

### Ami Client Application

**Figure 3:**
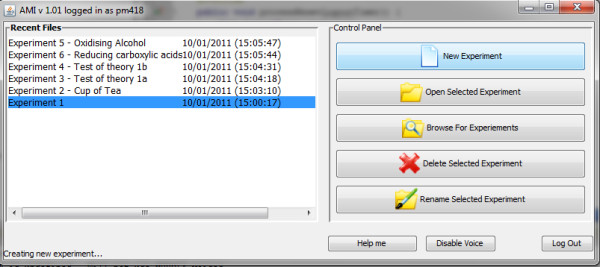
**The Ami experiment selection screen**.

The Ami client application - "Ami" - is the central application with which the chemist interacts when in the lab. The chemist logs into Ami using their identity badge which is detected by a Touch-A-Tag RFID reader. A list of experiments is then displayed, plus the option to create a new experiment (Figure 3).

Having selected their desired experiment, Ami then displays the main experiment control screen. Tabs at the top are used to switch between the Event Log, Sensor Control, and Experiment Details screens. All tabs and buttons can be controlled using speech, the keyboard, or the mouse.

**Figure 4:**
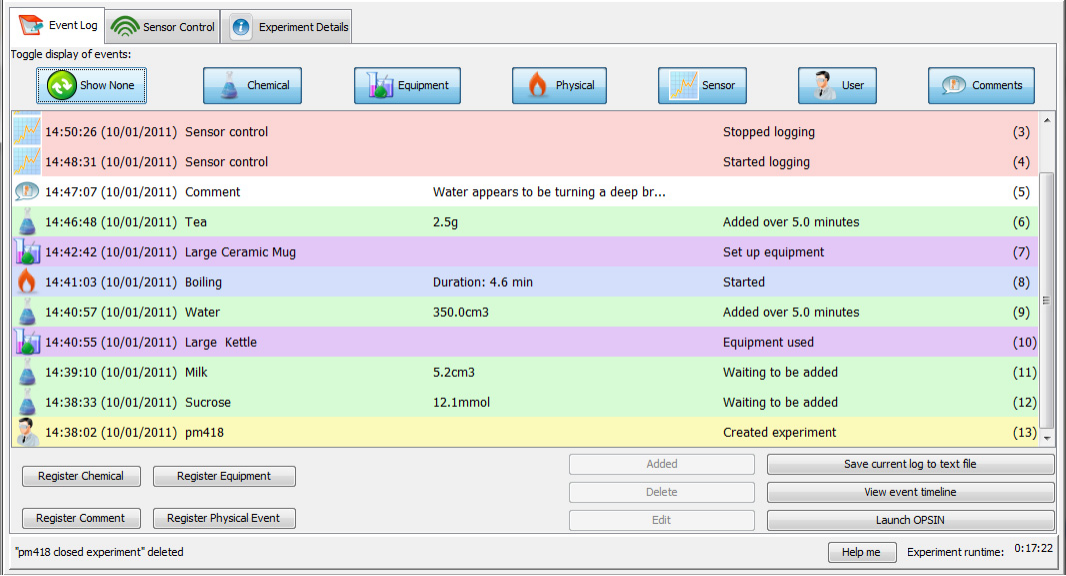
**The Ami Event Log screen**. The main body shows events logged. Buttons across the top allow selection of different event types. At bottom left are buttons to log different events. Buttons at bottom right are for saving data and launching the timeline viewer. Tabs at the top switch the display to the Sensor Control and Experiment Details screens.

The Event Log shows all the events that have occurred in the experiment. The log can be filtered by event type if desired (Figure 4).

Ami allows all chemicals and pieces of apparatus used in the experiment to be tagged with an RFID tag. These are easily and cheaply available in a variety of forms so that they are easy to stick to chemical bottles and apparatus. During the experiment, the chemist registers all the components with Ami.

As an experiment proceeds, the chemist logs usage of chemicals and apparatus by simply waving them in front of the RFID reader. The date and time of the event is recorded by Ami, so that a timeline of events in built up showing activity in the experiment. The chemist can also add observations by dictating to the PC's microphone, or simply by using the keyboard.

**Figure 5:**
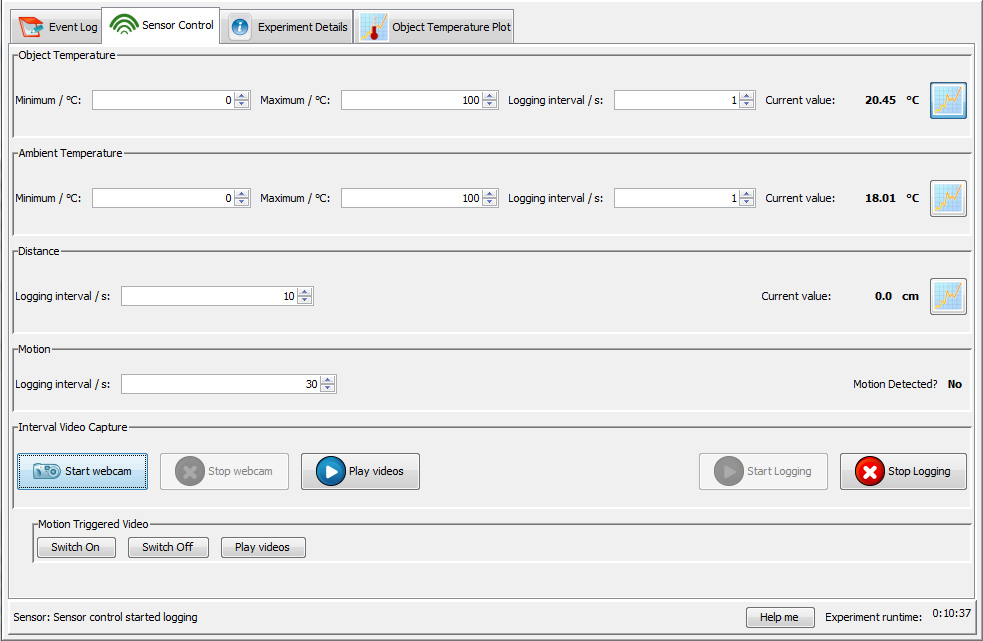
**Sensor control tab**. Alarm limits can be set for upper and lower temperatures. Time interval can be specified for taking temperature, distance (ultrasonic sensor), and motion detection (infrared motion detector). Buttons at bottom left are used for starting motion-timelapse video and environs-monitoring video. Buttons at top right control display of sensor graphs.

A refinement of the RFID tagging was an "intelligent labcoat". This was achieved by using a mini Arduino card with an RFID reader built into the sleeve. The Arduino had a Bluetooth transmitter with it, which was able to transmit readings to the Ami application running on a PC. The RFID detector in the labcoat sleeve automatically registers any tagged chemical or piece of equipment that the chemist's hand goes near. The events logged by the labcoat are then transmitted to the Ami application for including as part of the experiment timeline (Figure 5).

**Figure 6:**
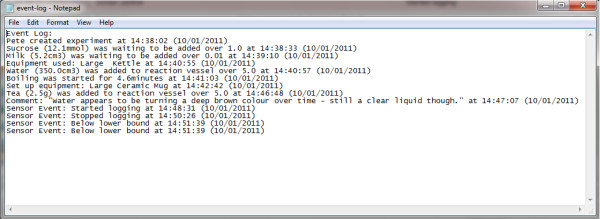
**Example event log file for the creation of a cup of tea, inspired by the Southampton Smart Tea project **[30]. New events are appended as they occur.

**Figure 7:**
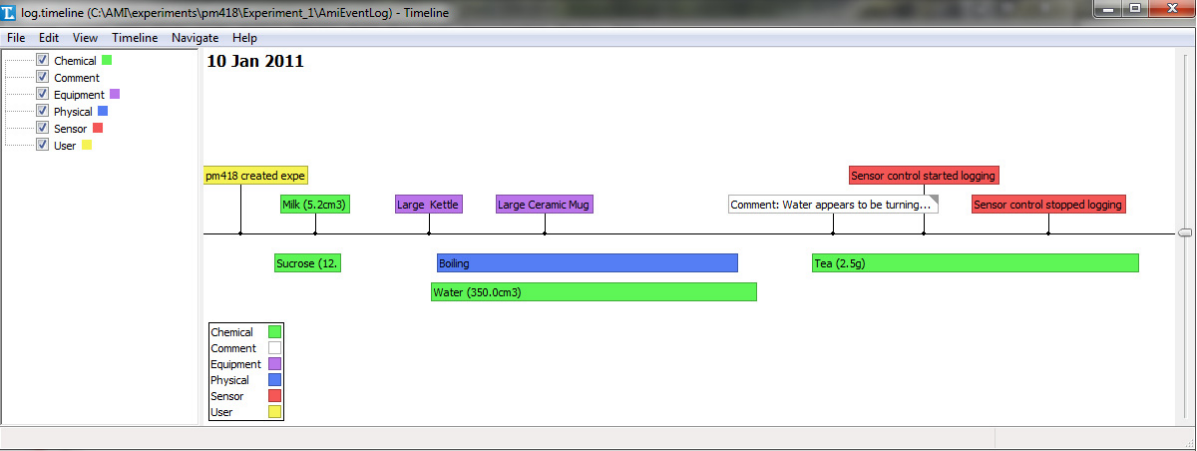
**Graphical view of experiment timeline**. The Timeline application [31] is written in Python and receives an XML event-log file created by Ami. Timeline takes this event log and displays different sorts of events in different colours.

Each sensor monitored by Ami has its own logfile (Figure 6). All output files are stored in one directory for each experiment, making it easy to keep track of all data created, and to transfer it to the electronic lab notebook. The output from all the sensors and events can be displayed as a graphical timeline to facilitate review of the experiment activities (Figure 7).

### Monitoring Device - Arduino

**Figure 8:**
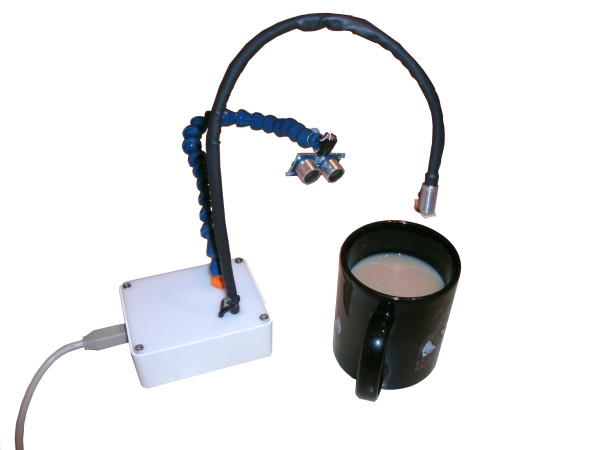
The Ami Experiment Monitoring Tool, here monitoring tea temperature...

**Figure 9:**
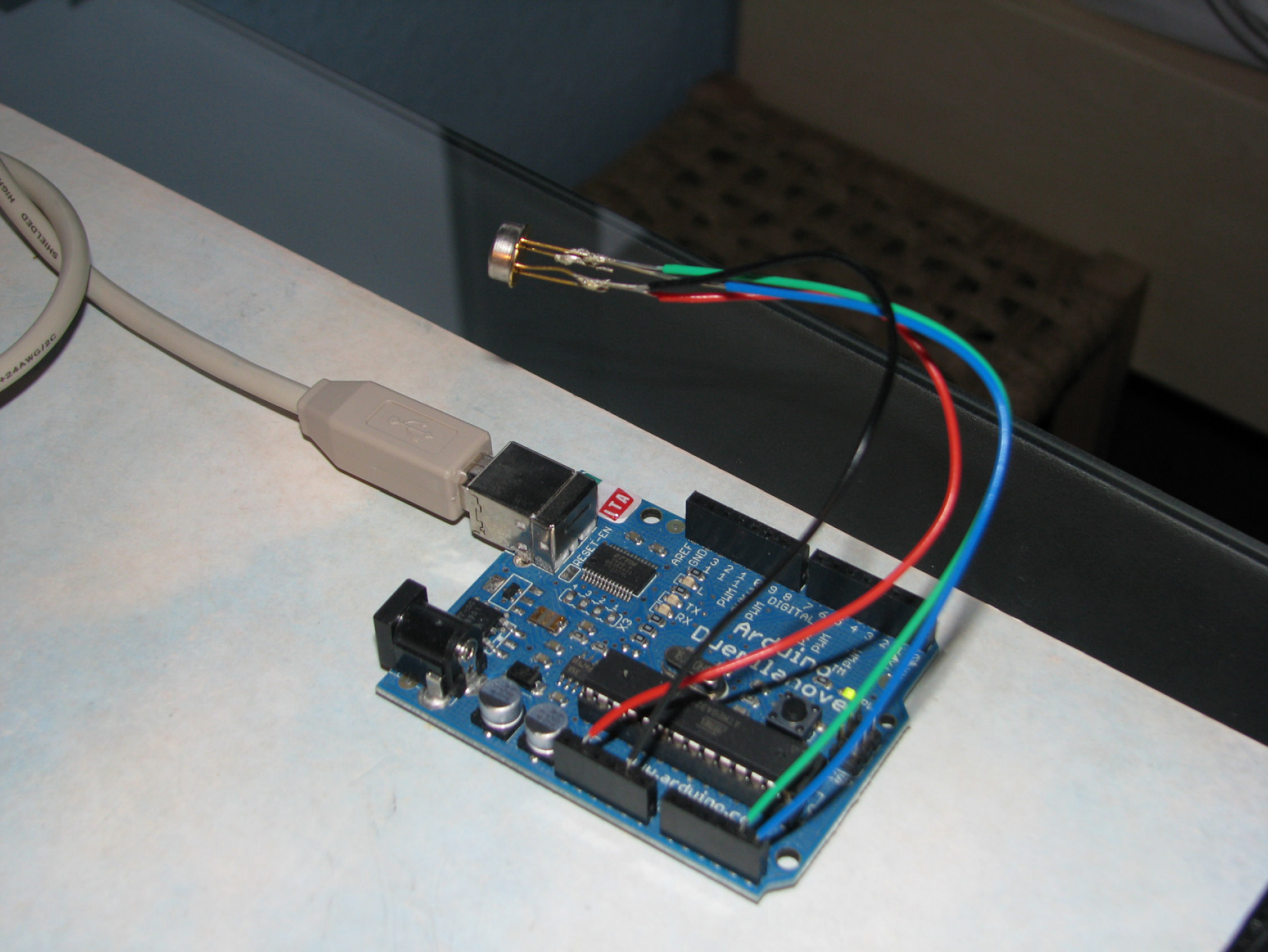
**The infrared sensor being tested on an Arduino circuit board**.

For close-up monitoring of the reaction, an infrared temperature sensor was used (Figure 8). This was controlled by an Arduino circuit board (Figure 9), which was programmed using the open source Arduino software [9].

The Ami Experiment Monitoring Tool also has an ultrasonic distance sensor and an infrared PIR motion sensor. Output from the sensors is sent to the Ami client application, which is a Java program running on the PC.

### Close-up video monitor

The usual way of doing time-lapse photography is to take a still picture at regular intervals, then stitch them together to make a moving picture. We wanted to do something slightly different; instead of a still picture, we wanted to use a few seconds of normal moving video, and then stitch them all together to make a time-lapse video. The advantage of this is that it is then possible to see how a given material is behaving (*e.g*. viscosity) which isn't possible from a still picture.

Recording time-lapse video turned out to be more difficult than expected. We were unable to find an off-the-shelf application that we could use to provide this functionality. Open source Java routines to monitor video had performance issues, for example very poor frame-rates. The main problem seemed to be that available Java-based open source code was out of date; it was all based on the Java Media Framework (JMF) [10], the API of which has not changed since 1999, and the last minor modification was in 2004. The open-source FFmpeg [11] utility was also tried, but its video capture functionality is provided through "Video For Windows" (V4W) which is not supported on Windows 7. Eventually we settled on VLC [12], which is based on Microsoft's DirectShow framework (better supported and up to date).

Because VLC is an application, the problem arose as to how to start and stop it from the Ami application. Fortunately VLC can be controlled via a telnet connection, so Ami uses telnet to configure the video capture and to start and stop video capture. There is an additional bonus that this separation of video recorder from controller enables multiple cameras to be used and also to start and stop recording on remote systems, without a physical connection to them.

Linking the videos together also turned out to be more difficult than expected. Modern compressed video formats such as AVI, MPEG4 work by encoding differences between successive frames. When concatenating video it is therefore necessary to decompress the videos before combining them together and re-encoding them using a given compression algorithm, such as an MPEG4 based codec.

Fortunately, VLC again has the ability to do this task but due to the nature of video concatenation, this process of stitching together the files is best done at the end of an experiment, rather than repeating the CPU-intensive process for each stage. Compression artefacts are extremely liable to arise from the process of repeatedly decompressing and recompressing as well, degrading the quality of the video. It may be possible to 'pause' a recording using the VLC capture, but if any errors arose or power is lost, the video data would have to be recovered manually.

Storing video files alongside the experimental data enables the logs to travel with the data, given a repository such as the ELN that can accept arbitrary files as part of a submission.

### Wide-angle video monitor

A common source of error in doing experiments is simply absent-mindedness, forgetting to do something, or using the wrong chemical. The wide-angle video monitor is triggered by activity at the fume cupboard, and records video until activity stops. This gives the ability to replay events over the course of the experiment, hopefully enabling a full picture of what actually was done to be understood. Mounting the webcam high up at the back or side of the fume cupboard gives the best view of activities.

Two methods of triggering the recording were identified. The first was to use an infrared movement detector, which was connected to the Arduino. When movement was detected, the event is passed by the Arduino back to the Ami program, which then starts the video monitor. The video simply records for a specified duration after movement is no longer detected. The second method was by monitoring the changes in the image itself, and if a threshold value is reached, to start videoing. Unfortunately time did not permit us to explore this area sufficiently to get a working system going.

### Experiments with the Microsoft Kinect

**Figure 10:**
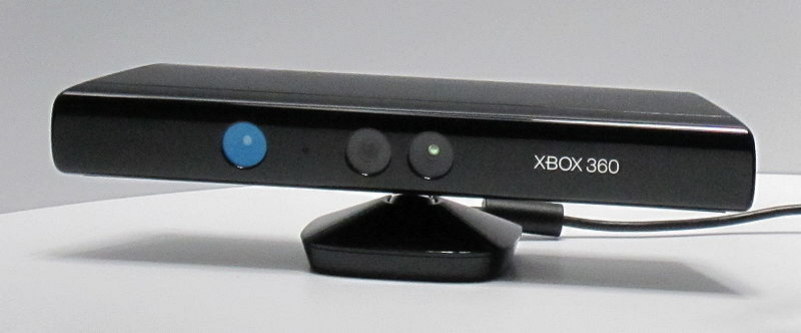
**The Microsoft Kinect**. This connects to a computer via a USB connector, making it a powerful way to communicate with computers. The infrared transmitter is on the left.

Towards the end of the Ami project, Microsoft released the Kinect (Figure 10). The Kinect is an accessory to Microsoft's Xbox consumer gaming machine, and is an exciting new development in human-computer interactions because it uses purely visual techniques to build a three-dimensional understanding of the space in front of it. The Kinect can recognise when a person is standing in front of it, and automatically determine the positions of the person's head, body, arms, hands, legs and feet. The user does not need such things as a transmitting device or special reflective tags; they just have to stand in front of the Kinect. The Kinect is potentially a disruptive technology and is already showing huge potential in robotics [13]. It has enormous potential for Ami; positioning a Kinect in a fume cupboard could give the user new ways to interact with the computer, and help in monitoring the environment.

The Kinect consists of a relatively small box (about 25 × 12 × 3 cm) which has two video cameras built into it [14,15]. One video camera is used for normal videoing using visible-light. The other is an infrared camera which monitors a pattern of infrared dots that the device shines into the room [16,17]. The on-board processing built into the Kinect enables it to understand the 3D location of all the objects in front of it (*i.e*. the spatial analysis is done by the Kinect itself, rather than by the computer that it is attached to). The attached computer receives from the Kinect a video feed plus a stream of data points of the 3D locations of all the objects detected by the Kinect. The Kinect also contains four microphones, but using these was not investigated in this project because at the time that this work was done no code had been released which made the sound output available.

One slight limitation of the Kinect is that its 3D view of the area in front of it is necessarily only seen from one position [18]. This means that it cannot understand a full three dimensional view of an object, because it can only see the side nearest the detector. Anything behind an object, and the back of an object, cannot be seen. This could be improved by using more than one Kinect operating together so that they can pool their individual views, and no doubt the techniques and code necessary to achieve this fuller 3D view will emerge over time [19].

#### Monitoring 3D space

The Kinect returns a three dimensional description of what it can see in front of it as well as a conventional 'RGB' view. The resolution of the normal colour image camera is 640 × 480, whereas the three dimensional camera is 320 × 240 [20]. Whilst this makes it a poor choice for image recognition, logging and so on, the depth camera delivers data that is fundamentally unavailable from other sources. This makes it incredibly exciting in terms of the types of data and interaction it can enable.

The working range of the Kinect is suitable for a large living room, as it was designed with that in mind. It was found that in the cramped confines of a fume cupboard the detector was not far enough away for reliable operation. This rather precludes the Kinect from being used for monitoring the 3D environment within the fume cupboard (the size of a typical fume cupboard is about 1.7 m wide × 1.2 m high × 0.7 m deep). So we turned our investigations to using the Kinect for controlling the computer itself; because the Kinect monitors body movements, it might be good for someone who is wearing protective clothing.

#### Using the Kinect to control a mouse

**Figure 11:**
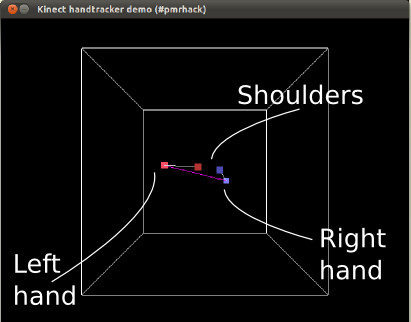
**Screenshot showing 3D visualisation of the Kinect's interpretation of the volume it can see**. Only the user's shoulder and hand joints are shown for clarity.

One of the intuitive ideas for using a Kinect is to detect human movement and gestures to control a computer, so called 'natural interaction'. One of the first experiments we tried involved detecting a hand and coupling a mouse pointer to respond to its movements. This was done at first by crudely detecting the closest 'blobs' present in the depth, i.e. the hands, and using the motions of these to control the mouse pointer of a computer. At a later stage, we used a much more sophisticated technique to capture the hand motions, which involved 'skeleton mapping' that understood and interpreted the depth of field and looking for humans (Figure 11). (Skeleton-mapping, as provided by PrimeSense [21], required a stack of software to enable, including the http://www.OpenNI.org framework and the SensorKinect driver [22]https://github.com/avin2/SensorKinect as well as their free closed-source middleware library.) This proved to be much more accurate and not affected unduly by background movement.

However, this mouse-metaphor interface proved to be a poor one in the end. This was not due to technical reasons; simply, the human body is not suited to standing still with an arm held out for periods of time. With a hand resting on a desk, it is easy to have the accuracy needed to click on items. With the arm stretched out, it becomes difficult to hold it in a given position for any amount of time.

It is necessary to build the interface so that the interactions involve periods of relaxation or the ability to ignore actions made unintentionally. One particularly successful form of interaction is selecting items from a menu, where the hand is raised to select an item from a list and a choice is made by swiping the hand across. Swiping the other hand back across is used to cancel that choice. This has the benefit that in between choosing, the arms can be left to relax without worrying that a mouse-pointer would skitter across the screen and select or highlight something unintentionally.

#### Using the Kinect to control molecule visualisation

**Figure 12:**
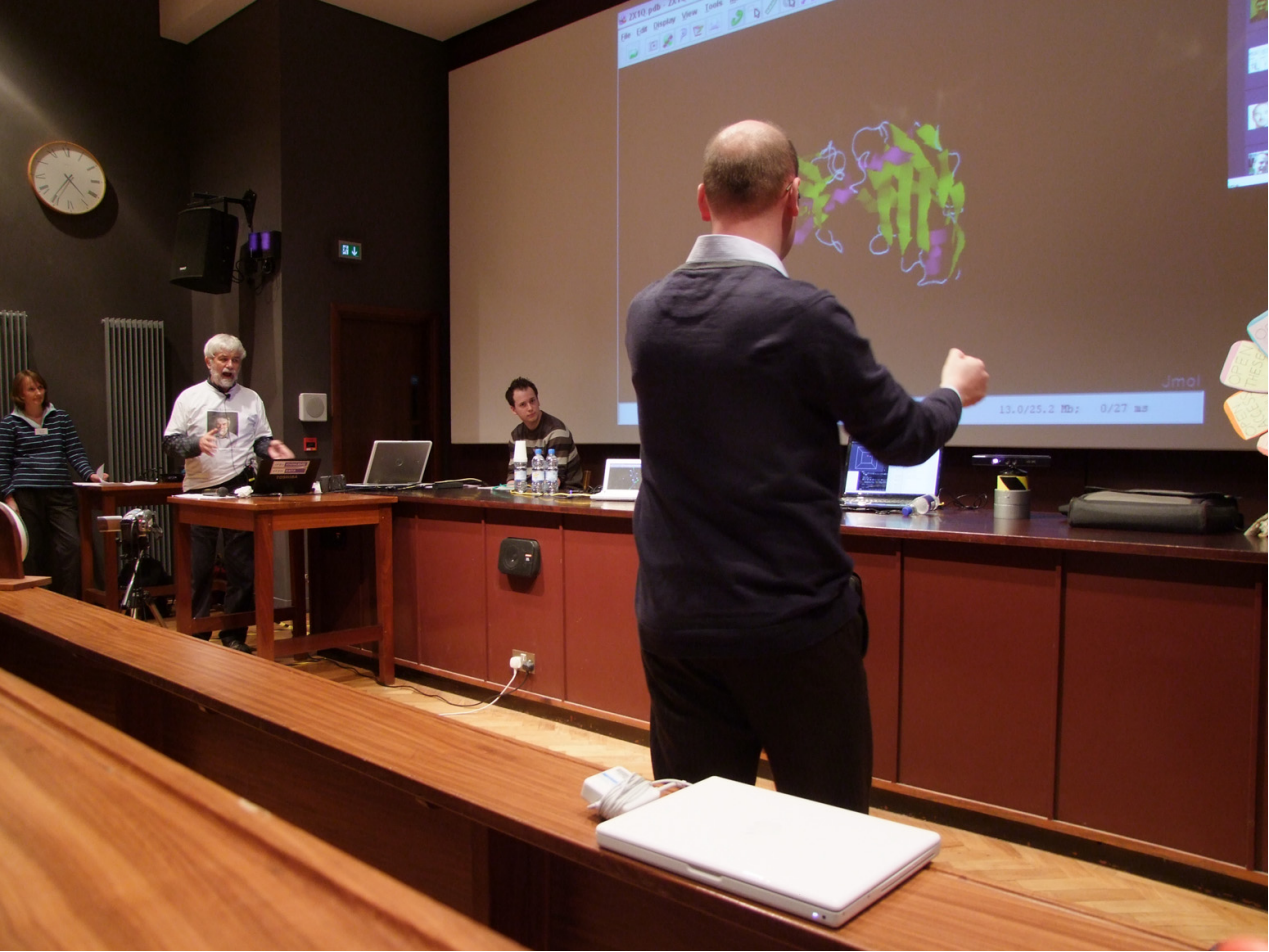
**Controlling rotation of a molecule using hand gestures**. The Kinect is on the bench, on top of the silver cylinder. Picture taken at PMR Symposium.

Moving away from the fume cupboard for a moment, one potential use-case for the Kinect is to use it as an appliance in a given meeting room or open space for collaboration or interaction. We implemented this by using the Kinect's skeletal mapping functions to track hand positions which were then broadcast via multicast to any computer within the same network. This has the advantage that the client computers need no knowledge of how to read and process Kinect data directly, only software that can make use of the hand-position mappings in the same way it might read the location of a mouse pointer [23]. At the end of the project, a symposium was held in honour of Dr Murray-Rust's ideas [24]. Immediately preceding the symposium was a hackfest at which our experiences working with the Kinect were used to control the rotation and zooming of a molecule in Jmol (Figure 12).

### Speech recognition

For a chemist working in the lab, the ability to use speech to communicate with their computer would be a great advantage. Preparative work before the start of Ami showed that Windows Speech Recognition (WSR - the speech recognition facilities build into Windows 7) could be used to control [25] the Chemistry add-in for Microsoft Word, Chem4Word [26]. Dragon Naturally Speaking (DNS) is the leading speech recognition package, so this was also evaluated.

Both WSR and DNS have the ability to define macros for navigating around the screen, clicking on buttons, etc. The ability of these tools to use speech to control an application was tested by trying to use only speech to operate the Chemistry Department's ELN, a Java-based application developed by IDBS [27]. However, neither WSR or DNS worked very well with Java applications; WSR in particular is much more functional with Windows-based applications because it has closer ties into the operating system's understanding of what objects are being displayed. Controlling the ELN was best achieved using send-key type instructions; if keyboard shortcuts did not exist for a particular activity, then this limited the possible actions.

Both WSR and DNS can be used to dictate text into Java applications. The demands of a specialist chemistry vocabulary are pretty stringent, however, so for chemistry dictation the transcription accuracy varies significantly. It is possible to train each package to improve voice recognition, but this is a potentially enormous topic and it was not done to any significant extent in this project. DNS has the ability to digest sample documents that the user gives in order to understand their particular vocabularies, but this was not explored beyond initial configuration.

For the Ami application WSR was chosen for doing further development, mainly because of licensing costs; DNS is very expensive. WSR has a free extension, Windows Speech Macros, which enables tailoring of the commands used when speech is recognised. This was fairly successful and it is possible to navigate all of the screens and buttons in the Ami application. WSR listens for key phrases, and then uses send-key instructions (most commonly an Alt- < single-key > code) to send key codes to buttons in the Ami application. Additionally, WSR can be used for dictating comments (experiment observations) directly into Ami, though we did not have the time to investigate its accuracy or ways of enhancing it and it was only used by the development team.

## Outcomes & Conclusions

The main outcome from the project was a demonstrator application that shows how experiments and the environment around them can be monitored using various sensors and video monitors. We had the stretch goal of actually having this used by real chemists for real experiments, but unfortunately time prevented us from polishing the system to a sufficient level to allow this.

At the launch meeting for the Dial-A-Molecule EPSRC Grand Challenge [28], a common theme that emerged was the need to have access to chemical data. Much of the data generated in laboratories does not get collected and made available in a form that other chemists can use. Time pressures mean that very often scientists do not get around to making their data available. The Ami project showed how there is huge potential for computers to help the bench chemist in their activities in the lab, and to make much of this information available for further use. In its six months Ami has investigated many technologies and ideas; an obvious follow-on to the project is to consolidate these ideas into a fully integrated tool that can be used in real laboratories. Additionally, there is much potential for further work on the flow of data from the experiment to electronic lab notebooks to an embargo management tool and thence to open repositories, thus facilitating re-use. Reviewers have pointed out how important this type of data will be for retrospective analysis, especially in cases of unexpected results or experimental reproducibility.

## Competing interests

The authors declare that they have no competing interests.

## Authors' contributions

BJB was the project leader/manager. He did the project's bureaucracy and organisation, guided the project's direction, and did much of the investigations into the speech. He also wrote the paper.

ALT did most of the programming for the Ami application, and investigated most of the software tools used, including investigations into video capture. He also worked on the Arduino development.

MS, PM and SC worked on Arduino hardware and software development. They also added detail to the project's use-cases, and integrated the Arduino development into the Ami application. They also did demonstrations of the project.

BOS worked on building the video capture for time-lapse video motion-snapshot monitoring. He also did the investigations into the use of the Arduino.

SEA developed software for driving the RFID reader system and advised on application development, testing environments, and troubleshooting. He also helped with the brainstorming session.

JAT configured the code management systems used by the project, and provided feedback, expertise and advice on the design of the application.

PMR was the principal investigator on the project giving advice, guidance, encouragement and enthusiasm. He participated in the brainstorming session, reviewed project reports and both contributed-to and edited this paper.

All authors have seen and approved the final paper.

## Appendix 1: Links to documentation, code resources, etc

Brainstorming session:

• Output from the brainstorming session: https://bitbucket.org/jat45/ami/downloads/Notes%20output%20from%20Ami%20brainstorming%20session%207May10.docx

Project website & tags:

• Project blog: http://amiproject.wordpress.com

• Project Wiki: http://bitbucket.org/bjb45/ami-project

• Project code: http://bitbucket.org/jat45/ami/

Software used:

• Java development - IntelliJIDEA Community Edition: http://www.jetbrains.com/idea/download/

• Speech Macros - Windows: http://code.msdn.microsoft.com/wsrmacros

• JFreeChart Java graph package: http://www.jfree.org/jfreechart/

• Timeline application: http://thetimelineproj.sourceforge.net/

• Natty - Java library for processing data/times: http://natty.joestelmach.com/

• Video capture - VLC: http://www.videolan.org/vlc/

Project code:

• Data logger - Arduino program: https://bitbucket.org/jat45/ami/src/096e6df85d58/arduinoControllerWithoutSD/

• Ami application: https://bitbucket.org/jat45/ami

• Experiments with the Kinect: https://github.com/benosteen/Kinect-tracking-code

Other tools used:

• Speech recognition - Dragon Naturally Speaking: http://nuance.co.uk/

• RFID reader - Touch-A-Tag: http://www.touchatag.com/
